# Research integrity in the era of artificial intelligence: Challenges and responses

**DOI:** 10.1097/MD.0000000000038811

**Published:** 2024-07-05

**Authors:** Ziyu Chen, Changye Chen, Guozhao Yang, Xiangpeng He, Xiaoxia Chi, Zhuoying Zeng, Xuhong Chen

**Affiliations:** a The First Affiliated Hospital of Shenzhen University, Shenzhen University, Shenzhen, China; b Chemical Analysis & Physical Testing Institute, Shenzhen Center for Disease Control and Prevention, Shenzhen, China.

**Keywords:** academic misconduct, artificial intelligence, research integrity, research regulation

## Abstract

The application of artificial intelligence (AI) technologies in scientific research has significantly enhanced efficiency and accuracy but also introduced new forms of academic misconduct, such as data fabrication and text plagiarism using AI algorithms. These practices jeopardize research integrity and can mislead scientific directions. This study addresses these challenges, underscoring the need for the academic community to strengthen ethical norms, enhance researcher qualifications, and establish rigorous review mechanisms. To ensure responsible and transparent research processes, we recommend the following specific key actions: Development and enforcement of comprehensive AI research integrity guidelines that include clear protocols for AI use in data analysis and publication, ensuring transparency and accountability in AI-assisted research. Implementation of mandatory AI ethics and integrity training for researchers, aimed at fostering an in-depth understanding of potential AI misuses and promoting ethical research practices. Establishment of international collaboration frameworks to facilitate the exchange of best practices and development of unified ethical standards for AI in research. Protecting research integrity is paramount for maintaining public trust in science, making these recommendations urgent for the scientific community consideration and action.

## 1. Introduction

The principle of research integrity, foundational to the ethical conduct and dissemination of scientific knowledge, commands adherence to the highest standards of honesty, accuracy, and transparency. It ensures the credibility and reproducibility of scientific findings, forming the bedrock upon which public trust in science is built. However, the landscape of scientific inquiry is undergoing a profound transformation, driven by the rapid advancement and integration of artificial intelligence (AI) technologies. This evolution, marked by significant strides in machine learning, deep learning, and natural language processing (NLP), has reshaped research methodologies, offering unparalleled efficiencies and insights.^[1–3]^

However, alongside these advancements, new ethical challenges and forms of academic misconduct have emerged, challenging the foundational tenets of research integrity. The proliferation of AI has introduced sophisticated means of data fabrication, falsification, and plagiarism, posing fresh dilemmas in the maintenance of ethical standards. High-profile cases of misconduct, facilitated by the misuse of AI tools, have spotlighted the vulnerabilities within current regulatory frameworks, underscoring the need for vigilance and proactive measures.^[4]^

In response to this evolving landscape, our study aims to critically examine the implications of AI technology on research integrity, identifying both the potential risks and opportunities it presents. We seek to foster a dialogue within the scientific community about the ethical use of AI in research, advocating for a balanced approach that harnesses the benefits of AI technologies while safeguarding against their misuse. Through comprehensive analysis and discussion, this paper endeavors to contribute to the development of robust strategies and guidelines that ensure the upholding of research integrity in the age of AI, laying the groundwork for sustainable scientific advancement that remains firmly rooted in ethical principles.^[5]^

## 2. Current state and applications of AI technology

### 2.1. Application of AI technology in scientific research and its latest developments

**Figure 1.**
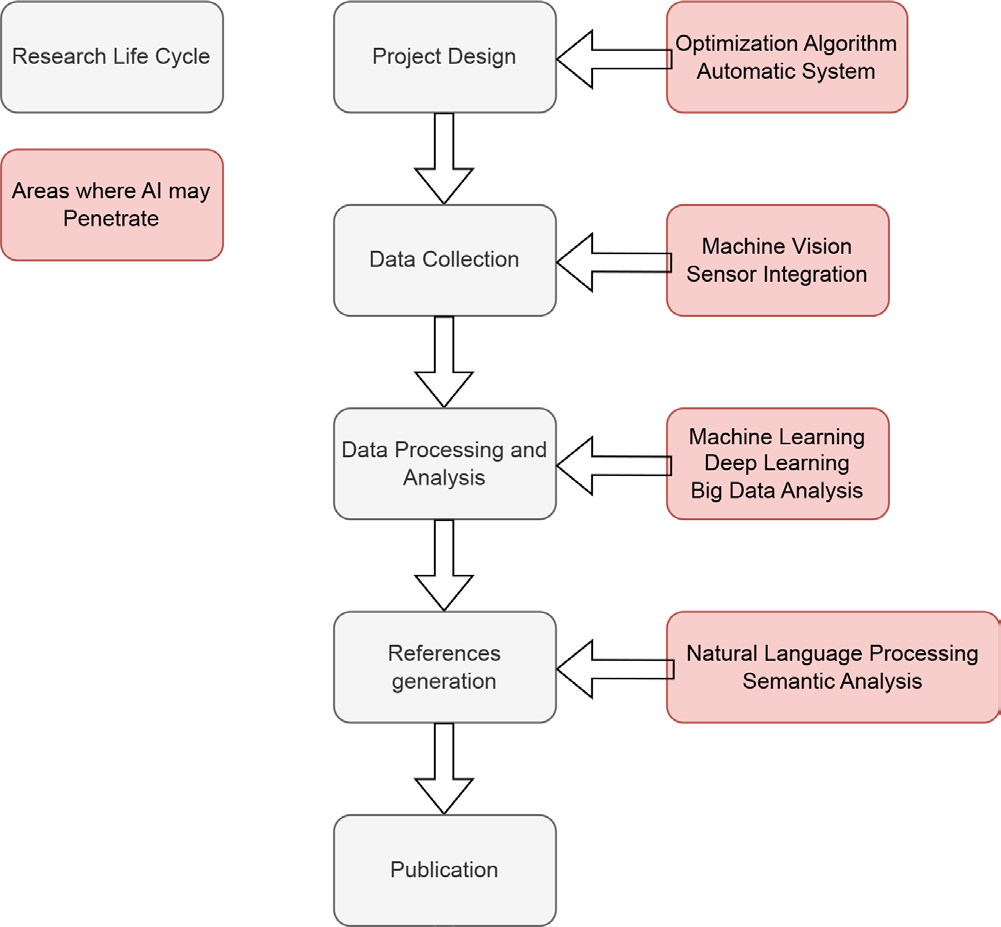
A visual concept map to outline the application of AI technology in the research lifecycle. Gray represents the research life cycle, and red represents the areas where AI may penetrate. AI = artificial intelligence.

Recent years have witnessed significant developments in AI, especially in deep learning, machine learning, and NLP. These advances have not only led to innovative algorithms but also enhanced computing power and data processing efficiency. AI role in scientific research spans from theoretical studies to experimental methodologies (Fig. 1).

Innovations in AI algorithms, cutting-edge research show advancements in AI models, especially in neural networks with complex architectures, offering improved data handling and predictive accuracy for unstructured data like images and texts.^[6–9]^

Genomic research breakthroughs, AI now plays a pivotal role in genomic research, going beyond data extraction to predicting gene functions and interactions. This evolution aids in understanding complex genetic diseases and tailoring medical treatments, as documented in recent biomedical literature.^[10–13]^ AI Impact on environmental science, machine learning is increasingly employed in environmental science, utilizing satellite imagery and sensor data to model climate change scenarios and monitor ecological changes with unprecedented precision.^[14–17]^ Materials science advancements, in materials science, AI accelerates the discovery of new materials with desired properties, reducing the reliance on extensive laboratory experimentation, as noted in contemporary research papers.^[18–20]^ Robotics integration, the fusion of AI with robotics is revolutionizing experimental research. AI-driven robots can autonomously conduct experiments, dynamically adjusting parameters based on real-time data, thereby enhancing experimental efficiency and reproducibility.^[21–24]^ Applications in social sciences, AI application extends to the social sciences, analyzing large-scale data to decipher patterns in human behavior. This informs public policy and economic strategies, as explored in recent social science research.^[25]^ Ethical considerations and governance, contemporary literature increasingly highlights ethical implications and the need for governance frameworks in AI applications, especially to safeguard data privacy and ensure responsible AI usage in sensitive fields like healthcare.^[26–29]^

These advancements underscore AI transformative role across scientific disciplines, presenting both immense potential and new challenges in research methodology and ethics.

### 2.2. The role of AI in data analysis, literature generation, and experimental design

In the realm of data analysis, AI technologies, particularly machine learning and deep learning algorithms, excel in processing big data, identifying data patterns, and conducting predictive analysis. For instance, by utilizing AI to analyze environmental data, researchers can predict climate change trends more accurately. This efficient data processing capability makes AI an indispensable tool in scientific research.^[30]^

The advancement of NLP technologies has also made AI pivotal in literature generation. AI tools, such as automatic summary generators and literature management software, assist researchers in efficiently organizing and summarizing vast amounts of scientific literature. This reduces the workload of literature reviews to some extent, allowing researchers to focus more on innovative studies.^[31]^

In experimental design, the application of AI technology significantly enhances both the efficiency and accuracy of experiments. Machine learning algorithms, for example, are used to design and optimize experimental procedures and predict outcomes. In some cases, AI can even automate experimental operations and data collection. This not only accelerates the experimental process but also improves the reliability of the results.^[32]^

These applications demonstrate that AI technology not only plays a supportive role in scientific work but has also become a key driver in advancing science. However, with the widespread application of AI technology, concerns have arisen regarding its potential to contribute to academic misconduct in research. This requires a joint effort from the scientific community and regulatory bodies to ensure the integrity and quality of research.^[32]^

## 3. Potential risks of academic misconduct in research

### 3.1. Data fabrication and falsification: How AI is used to generate or modify data

With the assistance of AI, data fabrication and falsification have become more sophisticated and difficult to detect. AI algorithms, especially deep learning models, can be used to generate realistic but entirely fictitious datasets. The application of this technology has already led to the retraction of several SCI papers, as it can generate convincing biomedical images and experimental results, posing a serious threat to the integrity of research. Fabricated data can not only mislead research outcomes but also lead to erroneous scientific theories and applications. For instance, a paper published in Physica Scripta on August 9, 2023, was retracted for this reason.

### 3.2. Text plagiarism and automatic content generation

AI technology, particularly sophisticated NLP systems, now has the capability to produce texts that seem to be original scientific content. These systems can automatically generate literature reviews, research reports, and even entire academic papers.^[31]^ The misuse of this technology could lead to a proliferation of pseudo-original content within the scientific community. This not only violates the copyright of the original authors but also potentially harms the authenticity and originality of scientific work.

### 3.3. Lack of transparency and disclosure: The issue of opacity in AI-assisted research

The application of AI technology in scientific research often lacks the necessary transparency. For instance, when AI algorithms are used for data processing or result generation, the working principles and decision-making processes of these algorithms may not be fully understood by researchers. This opacity can lead to misinterpretation and misuse of results. It has become an important issue in the scientific community to require researchers to provide comprehensive methodological descriptions and data processing details when employing AI technology.

### 3.4. Empirical analysis: Case studies of AI misuse

When examining the potential misuse of AI technology and its impact on research integrity, empirical case studies provide insightful and profound understandings. The following are several typical cases constructed from literature reviews and actual reports, demonstrating how AI can be misused in different contexts, posing threats to research integrity.

Generation of fictitious clinical trial data: AI technology can be used to fabricate clinical trial data supporting the efficacy of specific drugs. By employing advanced models to simulate real patient responses, these generated data may appear plausible on the surface but lack genuine experimental backing. Such practices not only mislead the direction of scientific research but could also have significant negative impacts on public health.^[33]^

Inappropriate application of statistical models: This involves the misuse of AI-generated statistical models in the field of environmental science. These models were applied without a full understanding of data characteristics, leading to incorrect predictions of environmental changes. This not only misguides related policy-making but can also mislead public perception of environmental issues.

AI-assisted academic plagiarism: Recent studies have indicated that AI technology is used to generate pseudo-original scientific papers. These texts, created by algorithmically reconstituting existing literature, appear to be unique content. Such plagiarism not only infringes upon the intellectual property rights of the original authors but also severely damages the integrity and originality of the academic community.^[34]^

Data fabrication and falsification: News reports have highlighted the improper use of AI in gene editing research, specifically in modifying experimental data to conceal adverse results or potential side effects. This manipulation of data directly affects the authenticity and credibility of research, thereby damaging public trust in scientific studies.

These cases highlight that while AI technology can enhance the efficiency and capabilities of scientific research, it also brings new risks of academic misconduct. Therefore, strict regulation and ethical guidance on the use of AI technology are crucial for maintaining research integrity and protecting the integrity of scientific research.

### 3.5. Taxonomy of academic misconduct in the AI era

As AI technologies increasingly permeate academic research, new forms and challenges of academic misconduct have emerged. To assist researchers and academic institutions in better identifying and mitigating these behaviors, this paper establishes a taxonomy of academic misconduct tailored for the AI era. This taxonomy categorizes misconduct based on type, perpetrator characteristics, severity, and common motivations.

(1) Type classification:

1. Data fabrication: This includes the use of AI to generate false data or manipulate data to conform to desired outcomes. Severity: High, common motivations: Publication pressure, pursuit of personal or team prestige.
2. Content plagiarism: Employing AI technologies for text auto-generation without proper citation or acknowledgments of original sources. Severity: medium to high, common motivations: Shortening research cycles, increasing output quantity.
3. Opacity of results: Utilizing AI for data processing and result generation without adequately disclosing methodologies or data sources, lacking replicability and verifiability. Severity: medium, common motivations: Protecting personal or team research advantages, technological secrecy.

(2) Characteristics of perpetrators:

1. Independent researchers: May resort to misconduct due to a lack of resources and oversight.
2. Research teams: Internal pressures and expectations can lead to misconduct by some or all team members.
3. Academic institutions: In extreme cases, institutions might indirectly encourage misconduct to maintain reputation or competitive standing.

(3) Interplay between severity and motivations:

This taxonomy further explores the relationship between the severity of misconduct and the motivations behind it, noting that high-pressure environments, intense competition, and an overemphasis on high output are common drivers of academic dishonesty. Additionally, the accessibility and ease of use of AI technologies lower the barrier to engaging in misconduct.

## 4. Detection and prevention of academic misconduct in research

### 4.1. Existing AI monitoring tools and techniques

As the complexity of academic misconduct escalates, particularly in the context of AI, a suite of monitoring tools and techniques has emerged to identify and mitigate these unethical practices. These innovations are instrumental in safeguarding the integrity of scientific research by ensuring the ethical use of AI technologies. The arsenal of tools encompasses:

Data integrity checkers: These tools scrutinize datasets for anomalies and inconsistencies, serving as crucial mechanisms to detect signs of data fabrication or falsification. By analyzing patterns within the data and benchmarking them against established norms, data integrity checkers flag discrepancies that may signify unethical manipulation, thereby preserving the authenticity and reliability of research data.

Plagiarism detection software: With the advent of AI, modern plagiarism detection software has significantly advanced to pinpoint AI-generated texts and pseudo-original content. Utilizing extensive databases for comparison, these platforms assess texts against a broad spectrum of sources to uncover potential instances of plagiarism, including sophisticated attempts where AI is employed to craft seemingly unique content.

Transparency and explainability tools for AI algorithms: These tools are designed to shed light on the opaque decision-making processes inherent to AI models, thereby promoting transparency in scientific research applications. By elucidating how AI algorithms interpret data and reach conclusions, they enable researchers and reviewers to comprehend and trust AI-assisted findings more deeply, fostering an environment where scientific discoveries can be critically assessed and validated.

Enhancements with data provenance and AI model auditing: Building upon the existing toolkit, this section introduces additional methodologies such as data provenance and AI model auditing, underscoring their pivotal roles in enhancing the transparency and traceability of AI research processes.

Data provenance: This technology traces the lifecycle of data, documenting its origins, movements, and transformations. Implementing data provenance tools ensures that datasets utilized in AI algorithms are accurate, ethically sourced, and reliably maintained throughout their use, thus reinforcing the credibility of research outcomes.

AI model auditing: Comprising systematic evaluations of AI algorithms, AI model auditing assesses their fairness, accuracy, transparency, and ethical implications. This process is essential for identifying biases, errors, or unethical components within AI systems, enabling corrective actions to align AI applications with ethical standards and regulatory guidelines.

These supplementary tools, including data provenance and AI model auditing, significantly contribute to the integrity of AI in academic research. By facilitating the transparent, accountable, and ethical deployment of AI technologies, they equip the academic community with robust mechanisms to navigate the ethical complexities posed by AI advancements.

### 4.2. Education and training: Enhancing researchers’ awareness of unethical AI practices

Education and training are key to preventing unethical AI-related practices in research. Recent studies and literature reviews have emphasized the importance of educating researchers on AI ethics and the recognition of unethical behaviors. This includes 2 main aspects:

Education on AI ethics and regulations: With the rapid development of AI technology, researchers need to be aware of and comply with the ethical standards and legal requirements when using AI. This includes, but is not limited to, data privacy protection, algorithmic transparency, and fairness. For example, educational programs could include courses on how to use medical data while protecting subject privacy, or workshops on preventing algorithmic biases and ensuring the fairness of research outcomes.

Training in data management and analysis: Proper data management and analysis are the cornerstones of research integrity. Training should cover how to effectively manage large datasets, apply appropriate statistical methods, and avoid common data analysis errors. These trainings can help researchers identify and prevent potential unethical data practices, such as selective data reporting or data tampering.

Additionally, as the application of AI technology in research continues to grow, educational and training programs also need to be continuously updated to reflect the latest technological developments and ethical challenges. This may include specialized workshops on emerging AI technologies (such as deep learning or NLP) and lectures on how to evaluate and report research results generated by AI. In summary, through these educational and training measures, researchers will better understand and address the ethical and regulatory requirements in research during the AI era, thereby helping to maintain research integrity and enhance the quality and reliability of their work.

Specific recommendations for educational and training programs include:

1. Offering a mandatory “AI Ethics and Regulations” course that covers AI ethical principles, relevant laws and regulations, case studies, and incorporates lectures, case discussions, and simulated practices.
2. Introducing elective courses such as “Big Data Management” and “AI-Assisted Statistical Analysis” in graduate and doctoral programs to cultivate relevant skills among students.
3. Organizing regular AI ethics seminars and workshops, inviting experts and scholars to share the latest developments and discuss related issues.
4. Encouraging interdisciplinary collaboration by involving experts from computer science, law, social sciences, and other fields in developing curricula and training content.
5. Establishing online self-study platforms that provide resources on AI ethics, data management, and other relevant topics for continuous learning by researchers.

By implementing these specific educational and training programs, we can systematically enhance researchers’ ethical awareness of AI, reinforce compliance, and promote the responsible application of AI technologies in academic research.

### 4.3. Formulation and implementation of policies and procedures related to research integrity

In maintaining research integrity in the era of AI, the formulation and implementation of relevant policies and procedures are particularly crucial. These measures are designed to ensure the integrity of research activities and the reliability of scientific outcomes, specifically including:

Development of a code of conduct for research activities: This involves establishing clear guidelines for behavior in research activities. Key aspects include data handling, result reporting, and author contributions, encompassing the assurance of data authenticity, transparency, and the accurate reporting of research findings. Establishment of review and oversight mechanisms: Research institutions should establish mechanisms to review research project applications and outcomes, ensuring all research complies with ethical and legal standards. Additionally, effective oversight mechanisms should be established to promptly identify and address any issues in research. Building a regulatory framework for research integrity: Develop and refine regulations and systems for research integrity, establishing a robust regulatory framework. This system should effectively detect and prevent unethical behaviors in research, such as data fabrication, plagiarism, and improper use of AI technology.

Implementing these policies and procedures can effectively detect, prevent, and penalize acts of misconduct in the field of research, upholding the integrity and quality of scientific work. This not only helps prevent the occurrence of unethical behavior but also provides timely solutions when issues arise.

## 5. Ethical and regulatory frameworks

### 5.1. Analysis of current research ethics standards and their applicability in the context of AI

At present, there are a series of established ethical norms and guidelines in the field of scientific research, aimed at ensuring the integrity and quality of research. However, with the rapid development and widespread application of AI technology, these existing norms may need to be reevaluated and adjusted in the context of new technological realities. Current standards may be insufficient in addressing issues unique to AI, such as data privacy, algorithmic bias, interpretability of results, and transparency. For instance, traditional data management guidelines may not fully address the challenges posed by big data processed by AI algorithms, especially when it involves personal privacy and sensitive information.

### 5.2. Exploring new guidelines and regulations for managing AI applications in scientific research

In light of the limitations of existing norms, the academic community and regulatory bodies are exploring and developing new guidelines and regulations to better manage the application of AI in scientific research. These new guidelines encompass aspects such as the transparency of AI algorithms, the lawful acquisition and processing of data, and the accuracy and reliability of AI research outcomes. A key issue under consideration is how to ensure the ethical use of AI algorithms and applications, preventing their misuse or adverse impacts on scientific research.

### 5.3. Variations in AI research integrity regulations across different countries/regions

Internationally, there are variations in the formulation and implementation of ethics and regulations related to AI in scientific research across different countries and regions.^[35]^ These differences reflect the varying levels of technological development, cultural values, and legal systems in each country. Some nations may have established more stringent norms in the ethics of AI research, while others might still be in the preliminary stages. The importance of international cooperation is undeniable for the entire academic community, especially in establishing universal standards and sharing best practices. Through international collaboration, global challenges posed by AI can be addressed more effectively, promoting the standardization of research integrity worldwide.

Overall, as AI technology continues to permeate and evolve within the field of scientific research, ethical and regulatory frameworks need to be continuously updated and refined to ensure the integrity, transparency, and responsibility of research work.

## 6. Future outlook and recommendations

### 6.1. Predicting future trends in AI technology and their impact on research integrity

The development of AI technology is accelerating rapidly, with its applications expanding continuously. In the coming years, AI technology is expected to make greater advancements in efficiency, accuracy, and autonomy. For example, deep learning and machine learning technologies will become more efficient and precise in handling more complex datasets and solving more intricate scientific problems. However, these advancements may also bring new challenges, particularly in ensuring research integrity. The high level of automation and complexity in AI could lead to new forms of unethical behavior in scientific research, such as algorithmic biases and misleading results.

### 6.2. Specific recommendations for improving the research environment and enhancing integrity

To address the challenges brought by AI technology in scientific research, the academic community needs to take proactive measures to improve the research environment and enhance research integrity. Here are some specific recommendations:

Strengthen ethical and regulatory education in AI technology: Researchers should receive in-depth education on AI ethics and regulations, especially regarding data management and algorithm transparency. This should include education on data privacy, algorithm bias, and intellectual property issues. For example, this education could be provided through workshops, online courses, and seminars, ensuring researchers understand and adhere to ethical standards in AI-driven research.

Establish multidisciplinary collaboration mechanisms: Encourage collaboration between computer science, ethics, law, and other scientific disciplines to jointly develop and implement ethical norms and regulations for AI research. Such interdisciplinary collaboration ensures that the developed norms are comprehensive and adapt to the constantly changing technological environment. For instance, specialized committees or working groups could be established to oversee and update these norms.

Enhance regulatory and review mechanisms: Strengthen the regulation of the application of AI in scientific research to ensure the reliability and integrity of research outcomes. This might include enhancing the review process of research funding to ensure supported projects comply with ethical standards, as well as establishing specialized review teams to oversee the use of AI technology and the reporting of research results. Additionally, the introduction of third-party audits and evaluations could increase the transparency of research projects.

### 6.3. Emphasizing the importance of research integrity in the AI era

Research integrity becomes particularly important in the era of AI. As the application of AI technology in scientific research increases, ensuring the authenticity, reliability, and transparency of research activities becomes more challenging yet more vital. Recent SCI papers emphasize that research integrity is not only the foundation of scientific research but also key to maintaining public trust and support for science. Therefore, establishing and maintaining an honest, responsible, and transparent research environment is crucial for the healthy development of the scientific community.

In summary, with the rapid development of AI technology in the field of scientific research, the scientific community in the future needs to place greater emphasis on research integrity and take effective measures to address the new challenges and risks that arise. We must remember, although AI is interesting and convenient, it is merely a tool, not an author.^[36]^

### 6.4. Strengths, weaknesses, opportunities, and threats (SWOT) analysis

**Figure 2.**
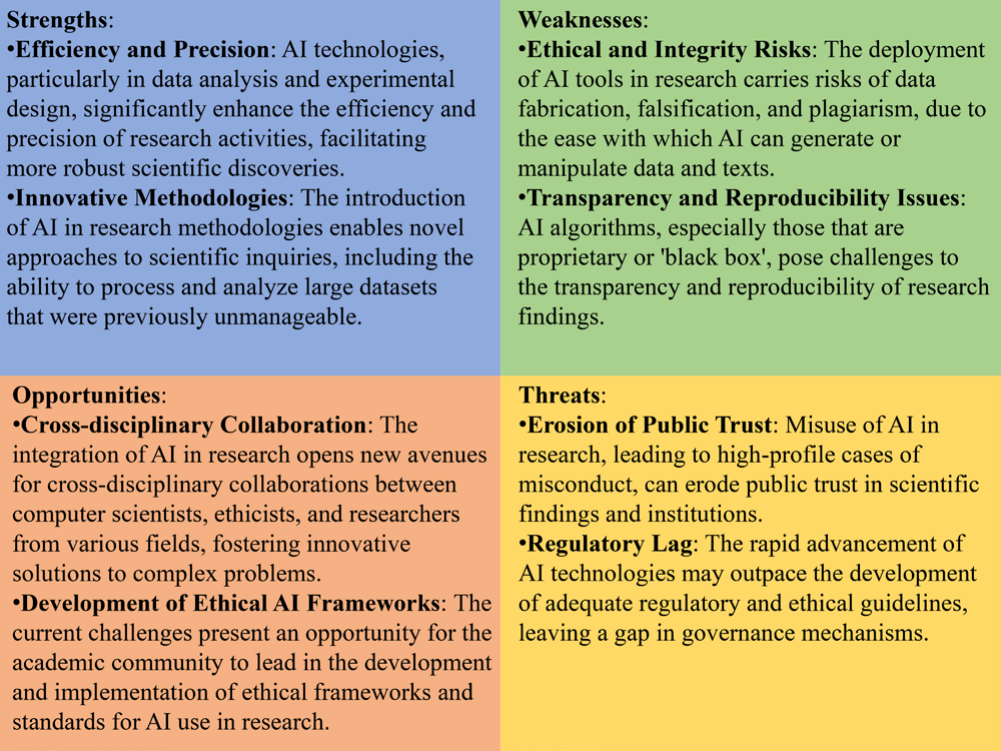
SWOT analysis of AI applications in academic research. This figure delineates the strengths, weaknesses, opportunities, and threats (SWOT) associated with the application of artificial intelligence (AI) technologies in the domain of academic research.

To systematically assess the multifaceted impacts of AI on research integrity, this study adopts a SWOT analysis framework. This methodology enables a holistic examination by categorizing the influences of AI into 4 distinct but interrelated domains: strengths, weaknesses, external opportunities, and threats. Each category is tailored to encapsulate the diverse dimensions through which AI intersects with the principles of ethical research. Utilizing the SWOT analysis, this study systematically evaluates these aspects through a review of current literature, case studies, and ethical discourse (Fig. 2).

## 7. Conclusions

The rapid advancement of AI technology has brought immense opportunities as well as challenges to the field of scientific research. While AI offers powerful tools to enhance efficiency, accuracy, and insights, its misuse or unregulated application poses new risks to research integrity.

This study has provided a comprehensive analysis of the impact of AI on academic misconduct in research. It has highlighted the potential threats, such as data fabrication, text plagiarism, and lack of transparency, which can undermine the authenticity and reliability of scientific outcomes. Empirical cases have been presented to illustrate the real-world manifestations of these issues.

To address these challenges, a multifaceted approach is necessary. Strengthening ethical norms, enhancing researcher qualifications through education and training, and establishing rigorous review mechanisms are crucial steps. Additionally, the development of new policies and regulations specific to AI applications in research is imperative to ensure responsible and transparent practices.

International cooperation is vital for establishing unified ethical standards and governance frameworks for AI in research. As AI technology continues to evolve, these frameworks must adapt to address emerging concerns and maintain public trust in scientific endeavors.

## Author contributions

**Conceptualization:** Xuhong Chen.

**Funding acquisition:** Zhuoying Zeng.

**Project administration:** Xuhong Chen.

**Writing – original draft:** Ziyu Chen, Changye Chen.

**Writing – review & editing:** Guozhao Yang, Xiangpeng He, Xiaoxia Chi, Zhuoying Zeng, Xuhong Chen.
